# Editorial: What makes leadership responsible and effective? Reinventing leadership in the COVID-19 outbreak

**DOI:** 10.3389/fpsyg.2023.1261313

**Published:** 2023-08-11

**Authors:** Fahmida Laghari, Samyia Safdar, Mastura Jaafar, Atasya Osmadi

**Affiliations:** ^1^School of Accounting, Xijing University, Xi'an, China; ^2^Department of Management Sciences, Capital University of Science & Technology, Islamabad, Pakistan; ^3^School of Housing, Building and Planning, University of Science Malaysia (USM), Penang, Malaysia

**Keywords:** responsible leadership, pandemic, COVID-19 outbreak, organizational psychology, organizational behavior

The pandemic of COVID-19 has been recognized as one of the most prevalent catastrophes of the recent period, and continued resilient leadership is needed to overcome the challenges it grants. As a result, diverse categories of leadership have taken the lead over reactions to concerns allied to COVID-19. In this way, leaders coped with the uncertainty of the crisis and offered new hope for future plans. As a result, people positively view the coronavirus, creating opportunities for progress and change.

This Research Topic entitled “*What makes leadership responsible and effective? Reinventing leadership in the COVID-19 outbreak*” introduces cutting-edge academic research and recognizes the fundamental role of leadership styles and theories in driving organizational psychology and organizational behavior theories and practices. The articles in the special issue examined a wide range of new leadership practices in organizations. We hope these articles will stimulate further research on leadership styles and theories and their application in practice. In this editorial, we offer several frameworks for thinking about styles and practices of leadership influencing organizational psychology and organizational behavior disciplines embedded with the notion of leadership, responsible leadership, and the effectiveness of responsible leadership in the COVID-19 outbreak, as shown in Figure 1. These frameworks organize the combination of articles in the special issue, identify potential gaps that merit further study, and present an agenda for future research.

**Figure 1:**
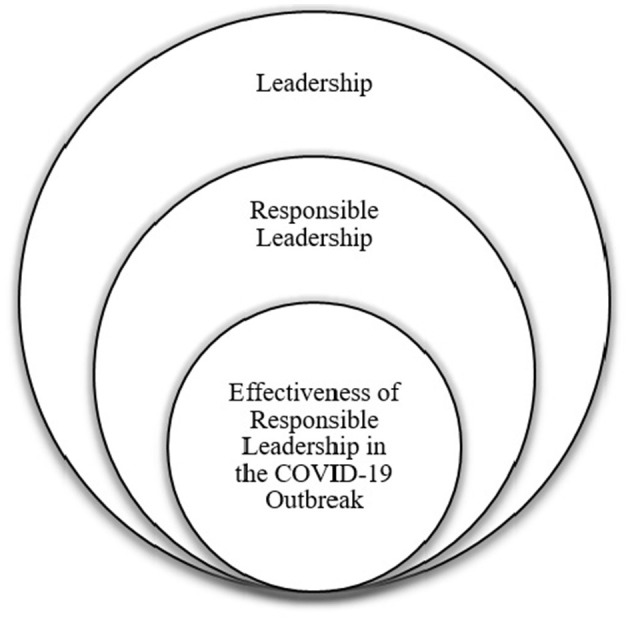
The research framework.

The prevailing research has defined leadership as how an individual or a set of individuals impacts others to attain a common goal (Ouchi, 1980). Leaders are often considered individuals with a unique grade of eminence or authority who can change the behavior of others to achieve mutually agreed-upon goals (Pfeffer, 1977). Equally, this prominence can be ceremonial or informal, and most of the views undertaken equally by the leaders and their followers consider that the leader has some form of genuine privilege over authority (Carton, 2022). Responsible leadership also comprises making coherent choices by emphasizing the aptitude to perform responsibly. The usage of customary leadership theory partially seizes this portent because other central extents of the leader-follower association may not be deliberated. Doh and Stumpf (2005) contend that responsible leadership is a cohesive style of authority, morals, and communal accountability.

As the notion of responsible leadership continues to evolve, so do the underlying concerns that require addressing and explaining over theory and research. Responsible leadership signifies a concept that happens at the juncture of two prevailing aspects of research, social responsibility, and leadership (Siegel, 2014). Though much has been inscribed about social responsibility, such as its link to a business's financial performance (Orlitzky et al., 2011), little is acknowledged about how people's activities and choices influence social responsibility. Similarly, Maak and Pless (2006) claim that responsible leadership is the community and moral exchange required to overcome several contests, though further researchers propose triangulating leadership, morals, and societal accountability.

In this special issue, there are nine related articles, which comprise manifold echelons and a collection of problems concerning the effectiveness of responsible leadership in the wake of the COVID-19 outbreak.

The first article by Zhang and Tian, with case study methodology, found that the trials they confronted established the supervision's closed management desires and the learners' demands for the liberty of entrance and departure, active and supple corrective growth, firm education assessment, big data-enabled authority, and the custom of social involvement-leaning management.

The second article by Qadri et al., with the PLS-SEM method, revealed that psychological contract breach (PBC) directly leads to emotional exhaustion (EH) and indirectly leads to job insecurity (JI) and organization distrust (OD) during the COVID-19 outbreak.

The third article by Kleynhans et al. revealed the impact of authentic leadership on employee flourishing and found that authentic leadership is a substantial predictor of employee flourishing over organizational support and organizational trust.

The fourth article by Aftab et al. examined how agile leadership plays its role in managing inter-role conflicts during the chaotic period of the COVID-19 pandemic. They found that agile leadership plays a substantial role in shaping job and life satisfaction.

The fifth article by Ye et al. investigated how online interaction in philanthropic marketing influences consumer impulse buying behaviors for products during the outbreak of the COVID-19 pandemic. Their study found that the herd effect of customers and the receptiveness of vendors could endorse customers' empathy for the cultivators of the goods vended livelily. The live streamers' image and the mutual trust between customers have a tiny influence on empathy promotions.

The sixth article by Lei et al. used SERVQUAL and CCSI models and explored the features that influence customers' satisfaction and loyalty. They found that popularity and credibility, delivery time commitment, and mailing security are the key issues that impact customer satisfaction and loyalty.

The seventh article by Xiong investigated the descriptive power of economic development and environmental audits on ecological environment quality. The study found that the amount of association and impact of environmental auditing on the ecological environment are higher than the amount of association and effect of economic development on the ecological environment.

The eighth article by Han et al. investigated the impact of uncertain expectations on the consumption behavior of rural residents in China. They found that consumers' income, consumption, consumption habits, liquidity constraints, and precautionary savings equally produce the spirits of doubt and will lead to countryside customers' indeterminate prospects.

The final article by Eid et al. suggests a theoretical roadmap for the research and teaching of local crisis leadership. They worked on complex problem-solving, team interaction, team context, technology, and team training design and declared that these four features denote significant obstacles if ignored.

In conclusion, we hope that this special issue of *Organizational Psychology*, a section of *Frontiers of Psychology*, stimulates more research on reinforcement learning by bridging the micro and macro divides in leadership practices, leadership effectiveness, leadership social responsibility, organizational psychology, and organizational behavior research. Responsible leadership effectiveness in the COVID-19 crisis is a significant topic to raise awareness of the causes and consequences of the effectiveness of responsible leadership in the COVID-19 pandemic actions. It also has an influential impact on corporate governance and managers' accountability to shareholders and other stakeholders, as well as on individual leaders, i.e., how reinforcement learning activities affect leaders' reputation and career development (personal interests). We must enlarge, define, and evaluate an expansive variety of vigorous strategies, and we need to better understand the role of collaborative governance in shaping vigorous problem-solving strategies (Ansell et al., 2021). Finally, the public policy implications of reinforcement learning are also prominent, as reinforcement learning addresses the behavior and effectiveness of responsible leadership in emerging and developed economies, the responses of these entities to organizational psychology, and its impact on reinventing leadership effectiveness in the COVID-19 outbreak to enhance organizational performance.

## Author contributions

FL: Conceptualization, Data curation, Investigation, Methodology, Project administration, Resources, Supervision, Validation, Visualization, Writing—original draft, Writing—review and editing. SS: Data curation, Investigation, Methodology, Project administration, Resources, Supervision, Validation, Writing—review and editing. MJ: Data curation, Investigation, Methodology, Project administration, Resources, Supervision, Validation, Writing—review and editing. AO: Data curation, Investigation, Methodology, Project administration, Resources, Supervision, Validation, Writing—review and editing.
